# Development and Evaluation of Superabsorbent Hydrogels Based on Natural Polymers

**DOI:** 10.3390/polym12102173

**Published:** 2020-09-23

**Authors:** Rejane A. Batista, Paula J. P. Espitia, Daviane M. C. Vergne, António A. Vicente, Paula A. C. Pereira, Miguel A. Cerqueira, José A. Teixeira, Jelena Jovanovic, Patricia Severino, Eliana B. Souto, Juliana C. Cardoso

**Affiliations:** 1 Post-Graduating Program in Biotechnology, Tiradentes University (UNIT), Av. Murilo Dantas, 300, Farolândia, Aracaju 49032-490, Brazil; rejaneabatista@gmail.com (R.A.B.); davianemcv@gmail.com (D.M.C.V.); pattypharma@gmail.com (P.S.); 2 Institute of Technology and Research of Sergipe (ITPS)—Rua Campo do Brito, 371—Bairro São José—CEP, Aracaju 49020-380, Brazil; 3 Nutrition and Dietetics School, University of Atlántico—Universidad del Atlantico, Puerto Colombia, Atlántico 081001, Colombia; perez.espitia@gmail.com; 4 CEB—Center of Biological Engineering, Universidade do Minho, Campus de Gualtar, 4710-057 Braga, Portugal; avicente@deb.uminho.pt (A.A.V.); paulapereira@deb.uminho.pt (P.A.C.P.); jateixeira@deb.uminho.pt (J.A.T.); 5 International Iberian Nanotechnology Laboratory, Av. Mestre José Veiga s/n, 4715-330 Braga, Portugal; miguelcerqueira@deb.uminho.pt; 6 Serbia Institute of General and Physical Chemistry, a.d., Studentski trg 12/V, 11158 Belgrade, Serbia; jelenajov2000@yahoo.com; 7 Institute of Technology and Research—Av. Murilo Dantas, 300, Farolândia, Aracaju 49032-490, Brazil; 8 Tiradentes Institute, 150 Mt Vernon St, Dorchester, MA 02125, USA; 9 Division of Engineering in Medicine, Department of Medicine, Harvard Medical School, Brigham and Women’s Hospital, Cambridge, MA 02139, USA; 10 Department of Pharmaceutical Technology, Faculty of Pharmacy, University of Coimbra, 3000-548 Coimbra, Portugal

**Keywords:** alginate, ionic complexation, xanthan gum, chitosan, hydrogel, superabsorbent polymer

## Abstract

Superabsorbent hydrogels (SAHs) are three dimensional networks formed by polymers that can absorb aqueous solution of over 100% of their initial weight. This work aimed to develop and characterize SAHs of Chitosan/Xanthan gum (CG), Chitosan/Alginate (CA) and controlled Chitosan (C), Xanthan gum (G), and Alginate (A) produced using “onion-like” methodology. The swelling performance, the morphological structure, the crystallinity, and the Fourier transformed infrared spectroscopy characteristics of SAH were used for the characterization of polyelectrolytes complex. Swelling analysis showed that chitosan has a strong influence on the maintenance of hydrogels structure after swelling, mainly in the acid environment (pH = 2). The chitosan hydrogel presented around 3000% of acidic fluid absorption after 24 h. The chitosan:xanthan gum (1:1 and 2:1 named as C1G1 and C2G1, respectively) hydrogels were the best combination regarding swelling performance in an acid environment, reaching 1665% and 2024%, respectively, as well at pH 7.0, presenting 1005% (C1G1) and 667% (C2G1). Scanning electron microscopy analysis showed samples with pores, and with different shapes. The X-ray diffraction showed the presence of a characteristic peak at 2θ = 20° in all developed composition because of the crystalline nature of chitosan. This work shows the possibility of developing eco-friendly biopolymer-based SAHs at a low cost with a good swelling capacity and stability.

## 1. Introduction

Superabsorbent hydrogels (SAHs) are hydrophilic systems formed by three-dimensional networks based on hydrophilic polymers capable of absorbing and retaining a high amount of water or aqueous solutions. They can be formed by two or more polymers linked among them physically (e.g., ionic bonds or hydrogen bonds) or chemically (e.g., covalent bonds) [1,2]. Generally, SAHs are characterized for the ability to absorb a high amount of water and other fluids (>100% their weight) in a short time and retain significant amounts of fluids in their internal structure [3]. Furthermore, SAHs maintain their three-dimensional structure after they are swollen and submitted to pressure [4,5]. The swelling potential of SAH, in terms of both swelling kinetics and equilibrium degree of swelling, depends on the primary structural properties of SAH that include many factors, such as the type of monomers, network structure, crosslinking ratio, degree of ionization of functional groups, and porosity. The swelling ability is also a function of the properties of the swelling medium, including the pH, temperature, ionic strength, or certain chemical species [6,7].

SAHs have been widely applied in personal care products. In this context, the commercial application of personal care products is wide, corresponding to 80% of the total hydrogel production [3]. Moreover, they have presented potential application in other fields, such as agriculture, waste water treatment, water purification, tissue engineering, sensors, contact lenses, and drug release [1].

Currently, most SAHs commercially available are prepared based on synthetic polymers derived from petroleum, which are nonbiodegradable, and nonecofriendly. Some polymers universally used in hydrogel elaboration are the ones based on acrylic acid, methacrylic acid, maleic acid, among others. Hydrogels of acrylic polymers and copolymers are known to exhibit adjustable swelling kinetics with specific properties, and the presence of poly(acrylic acid) (PAA) segments in SAH significantly increases their ability to swell in water [8]. The swelling kinetics of structurally different PAA hydrogels in bidistilled water at different temperatures and the effects of the pH value of the swelling medium on the PAA hydrogel, as well as the comparison of the swelling kinetics in water and physiological solution were thoroughly investigated [9]. Different approaches in the evaluation of poly(acrylic acid) hydrogel swelling are also available [10].

**Figure 1:**
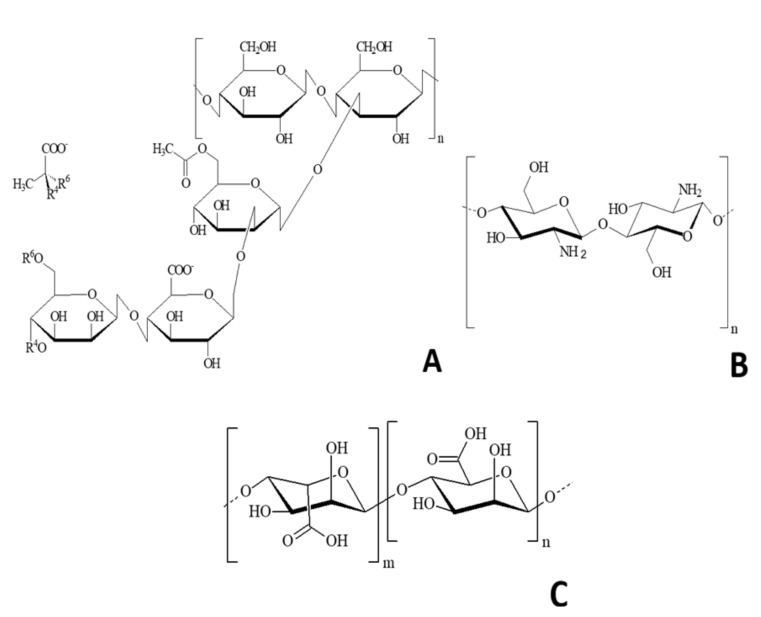
Schematic representation of: (**A**) Xanthan gum repetitive units; (**B**) Chitosan; (**C**) Alginate monomer.

The use of biopolymers obtained from renewable sources, characterized by their biodegradability, have emerged as an interesting alternative regarding the development of superabsorbent hydrogels, considering the environmental limitations of the synthetic materials. The advantages of using biopolymers for the development of SAHs include their biodegradability, compatibility with the environment, and low cost [4,11]. One interesting biopolymer with potential use in the development of SAH is the xanthan gum (G). This is an extracellular polysaccharide produced by *Xanthomonas campestris* by the fermentation process of industrial waste. Traditionally, this biopolymer had an important role in the food industry, and is used as thickener, emulsifier, and stabilizer [12]. G is a polyanion, in which carboxylic groups interact with amino groups of chitosan, forming an ionic complex due to differences in the ionic charge. G is structurally constituted by cellobiose as repetitive units and lateral chains are formed by the sequence of mannose, glucuronic acid, and mannose, which are linked to alternated glucose residues [12] (Figure 1A). Another natural polysaccharide is chitosan. This is biodegradable, cationic, and produced by the basic deacetylation of chitin (e.g., bioproduct by crabs and shrimp) (Figure 1B). This polymer presents properties generated from its amino groups, which have positive charges when dissolved at pH values below 6.5. This cationic nature allows the interaction of chitosan with other negatively charged compounds, such as enzymes, cells, polysaccharides, and nuclei acids [11]. Chitosan is a key biomaterial to obtain SAH, since it has excellent biocompatibility properties, and chemical characteristics that allow the high absorption of aqueous solutions. Moreover, alginate, a polysaccharide found in the cell wall of brown algae, also have potential to be used in the development of SAHs. Alginate, such as *Macrocystis pyrifera* and *Ascophyllum nodosum*, has been previously used in the food industry for preparing canned vegetables, marmalades, cakes, ice cream, cookies, among other products. Similarly, the gels formed from alginates can achieve the formation of organized molecular associations, making from this polymer a unique compound among gelling agents. The formation of hydrogels from alginate is produced by the ionic gelation, resulted from the crosslinking of alginate’s basic units (negatively charged) with multivalent cations (Ca^2+^, Zn^2+^, Fe^2+/3+^, Co^2+^, Ba^2+^, Al^3+^), e.g., interaction among the carboxylic ions of alginate and amino groups of chitosan [13]. Alginates (Figure 1C) are constituted by linear chains of polymers constituted by monomers of the mannuronic acid and the glucuronic acid, both linked by α1-4 bonds [14].

Although several studies are focusing on the effect of biopolymeric blends, publications regarding this topic are still limited. Moreover, innovative physical methods for the production of SAHs should be explored since it has been previously indicated that although chemical methods have a better relationship cost/benefit, they can result in harm to the environment. On the other hand, physical methods for SAH have been established as environmentally friendly; however, they are usually more complex and result in higher costs [15]. Thus, strategies to overcome the difficulties of producing eco-friendly SAH should be studied.

The development of biodegradable and at the same time stable SAH after swell is a challenge. The use of a high pH, “onion-like” methodology in combination with natural polymers seems to be an interesting strategy to overcome this challenge [16]. Therefore, this work aimed to develop SAHs based on a polyelectrolytes complex, using the “onion-like” method. The SAHs were evaluated regarding their swelling capacity and water contact angle; SEM, XRD, and FTIR were used to understand the morphology and the chemical changes during SAHs development.

## 2. Material and Methods

### 2.1. Materials

Chitosan (296.6 kDa of molar mass) was obtained from Polymer (São Paulo, Brazil). Alginate (150–170 Da of molecular mass) was obtained from FMC Biopolymer (Philadelphia, PA, USA). Xanthan gum was purchased from Sigma Aldrich (Deisenhofen, Germany). Double distilled water was used after filtration in a Millipore^®^ system (home supplied). All other reagents were purchased by Sigma-Aldrich (Sintra, Portugal).

### 2.2. SAH Preparation

**Figure 2:**
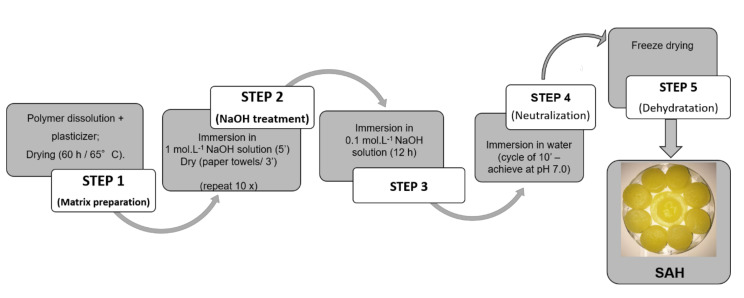
Flowchart of “Onion-like” process to obtain polyelectrolyte complex superabsorbent hydrogels (SAHs).

The polyelectrolytes complexes were prepared in different steps as described in Figure 2. The formulations were developed using different proportions of polysaccharides as shown in Table 1. Chitosan was dissolved in 2% lactic acid solution. The alginate or xanthan gum was dissolved in distilled water. The polymers were kept separately under mechanical shaking at room temperature (25 ± 1 °C) for 24 h. Then, the polymeric dispersions (chitosan and one of anionic polymeric dispersion) were mixed and shaken for 24 h more at room temperature (25 ± 1 °C). After this, glycerol (40% w/v) was added in dispersion. The dispersions (3 mL) were cast in Petri dishes and dried in an oven for 60 h at 65 ± 1 °C.

The second step, known as the “onion-like” methodology, was performed according to Ladet et al. [16] with modifications. Dried sponges were immersed in NaOH solution (1 mol·L^−1^) for 5 min, then dried for 3 min with filter paper. This process was repeated 10 times. After this, each formulation (Table 1) was immersed in NaOH solution (0.1 mol·L^−1^) for 12 h, and washed with distilled water until neutralization. The samples were then freeze-dried (Figure 2).

### 2.3. Swelling Degree

The kinetic of the swelling degree of developed SAH was determined according to Liu et al. [17] with some modifications. Samples with a 0.6 mm diameter (triplicate) were weighted and immersed in water (pH 7.0) or Tris-HCl buffer solution (pH 2.0). At different time intervals, the excess of solution at the surface was removed from the samples with a filter paper, and the samples’ weight was determined. This process was performed until the samples reached equilibrium (constant weight). The water absorption (α), or swelling degree, was calculated according to Equation (1).

(1) $$\alpha =\frac{\left(weight\;of\;hydrated\;film-\;weight\;of\;dried\;film\right)}{weight\;of\;dried\;film}\;\times \;100$$

### 2.4. Scanning Electron Microscope (SEM)

The morphology of developed SAH was characterized using a scanning electron microscope (FEI Nova Nano SEM 200, Hillsboro, OR, United States). Samples of SAH were cut (0.2 × 0.4 mm), placed in aluminum stubs, and were sputter-coated with colloidal gold particles (10 nm thickness) and then left drying before scanning. Sample observation was performed with an acceleration voltage of 10 kV under vacuum conditions.

### 2.5. Contact Angle Measurement

Deionized water (Millipore Milli-Q system) was used for the contact angle measurements. The deionized water was placed in a syringe (500 µL, Hamilton, Giarmata, Timis County, Romania), provided with a needle of 0.75 mm diameter. The water drop was released on the surface of each sample using a volume dose of 5 µL, at a rate of 2 µL/s. The contact angle formed by the water drop on the surface of the developed SAH was measured using a goniometer (OCA 20, DataPhysics Instruments GmbH, Filderstadt, Germany). Contact angle measurement was performed 30 s after the water drop release to avoid variations by solvent penetration in the samples. The assay was repeated 5 times for each formulation at 22.5 ± 0.5 °C.

### 2.6. X-ray Diffraction

The crystalline structure of SAH was accessed by X-ray diffraction analysis. The analysis was performed in an X-ray diffractometer (Bruker D8 Discover, Ettlingen, Germany), using Cu radiation (λ = 1.5406 Å). The Cu radiation was generated at 25 mA and 35 kV. The diffraction angle ranged from 5° to 50° (2θ).

### 2.7. Fourier Transform Infrared Spectroscopy

Fourier transform infrared (FTIR) spectra of SAH were recorded between 4000 and 400 cm^−1^, using a Shimadzu FTIR-8300 spectrometer (Shimadzu, Tokyo, Japan) at 25 °C. The resolution of the spectra was 8 cm^−1^.

### 2.8. Statistical Analysis

Analysis for swelling degree was performed in triplicate. Obtained data were submitted to ANOVA and a Tukey test at 5% probability using the statistical software Prism GraphPad 8^®^.

## 3. Results

### SAH Characterization

**Figure 3:**
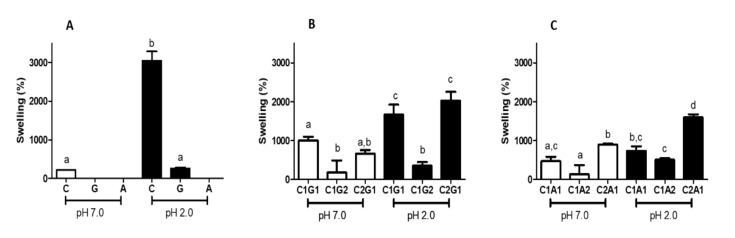
Swelling degree after 24 h of hydrogel polymers (**A**), polyelectrolytes complexes of chitosan and xanthan gum (**B**), and chitosan and alginate (**C**). The environment solution was distilled water (pH 7.0—white bars) and HCl-tris buffer (pH 2.0—black bars). C—Chitosan; G—Xanthan gum; A—Alginate; C1G1—Chitosan: Xanthan gum 1:1; C1G2—Chitosan:Xanthan gum 1:2; C2G1—Chitosan:Xanthan gum 2:1; C1A1—Chitosan:Alginate 1:1; C1A2—Chitosan:Alginate 1:2; C2A1—Chitosan:Alginate 2:1. Values with different letters are significantly different (*p* < 0.05).

The samples xanthan gum (G) and alginate (A) produced using the “onion-like” methodology did not keep a hydrogel structure after swelling in distilled water (Figure 3A). In the same pH, the samples obtained with the lower amount of chitosan presented a swelling degree lower than 200% after 24 h (C1G2—180% and C1A2—136%; Figure 3B,C, respectively). At acid pH, the highest swelling degree results were for C (achieving 3046%, Figure 3A), followed by C1G1, C2G1 (1665% and 2024%, respectively—Figure 3B), and C2A1 (1592%—Figure 3C). In both environments, C1G1 and C2G1 (Figure 3B) reached a swelling degree higher than 660%.

**Figure 4:**
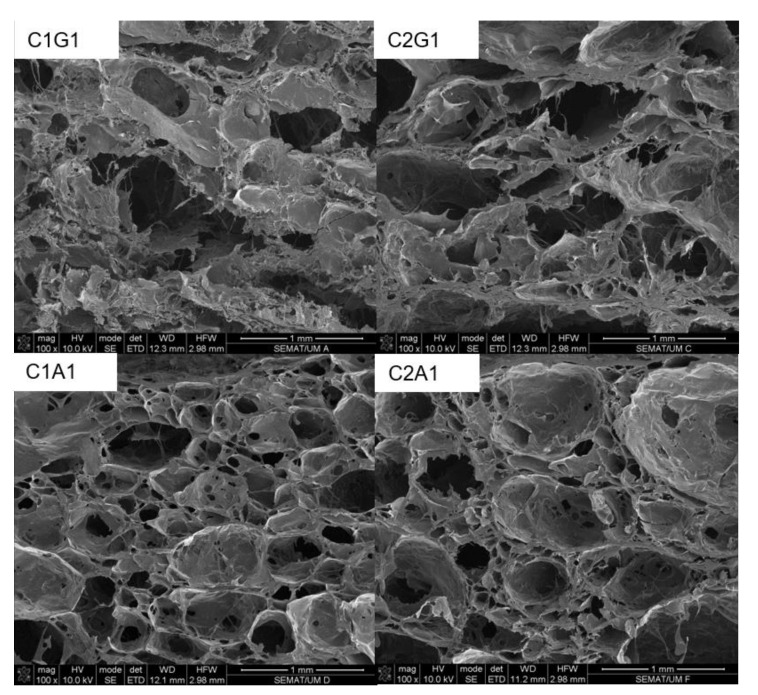
SEM images of superabsorbent hydrogels at different concentrations of biopolymers based on polyelectrolytes complexes. C1G1—Chitosan:Xanthan gum 1:1; C2G1—Chitosan:Xanthan gum 2:1; C1A1—Chitosan:Alginate 1:1; C2A1—Chitosan:Alginate 2:1.

In Figure 4, it can be observed the formation of pores in the inner structure of all samples that presented higher swelling degrees (i.e., C1G1, C2G1, C1A1 and C2A1). The samples containing xanthan gum (C1G1and C2G1) presented irregular porous structure, while the sample containing alginate (C1A1 and C2A1) showed regular porosity.

**Figure 5:**
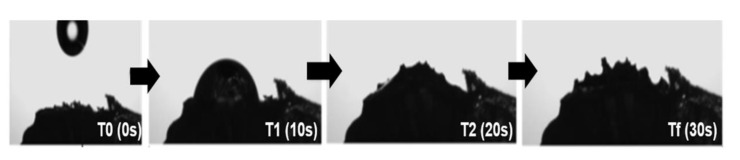
Hydrogel swelling process (images from goniometer assay using Xanthan-Chitosan hydrogels and distilled water). Source: Batista et al. (2019) with permission [18].

In the contact angle determination, it was not possible to record the precise values during the measurement. Before dripping, a projection of the sample surface toward the drop of water was shown, probably because of the high affinity between the hydrogel surface and water. However, by using the camera coupled to the goniometer, it was possible to capture the sequence of images that represent schematically the moment when the water drop was absorbed by the developed hydrogel. This behavior was observed in all formulations (Figure 5).

**Figure 6:**
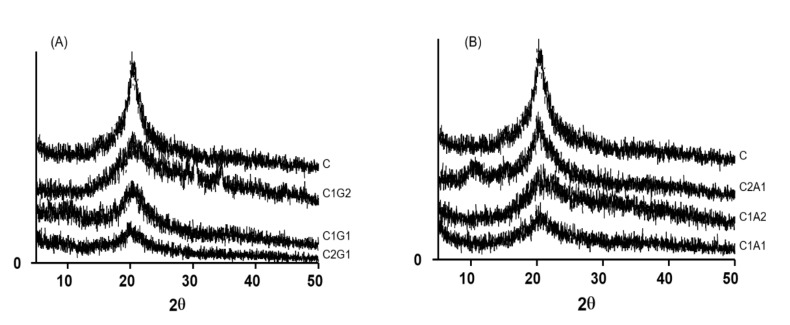
X-ray diffraction patterns of chitosan and polyelectrolyte complexes SAH, based on chitosan/xanthan gum (**A**) and chitosan/alginate (**B**). C—Chitosan; C1G1—Chitosan:Xanthan gum 1:1; C1G2—Chitosan:Xanthan gum 1:2; C2G1—Chitosan:Xanthan gum 2:1; C1A1—Chitosan:Alginate 1:1; C1A2—Chitosan:Alginate 1:2; C2A1—Chitosan:Alginate 2:1.

Figure 6A,B present the X-ray diffraction of the SHA based on chitosan/xanthan and chitosan/alginate, respectively. Results showed the presence of a characteristic peak around 2θ = 20° in all formulations. In samples with a high proportion of xanthan gum (C1G2) or alginate (C1A2), the peak at 2θ = 20° became broader.

**Figure 7:**
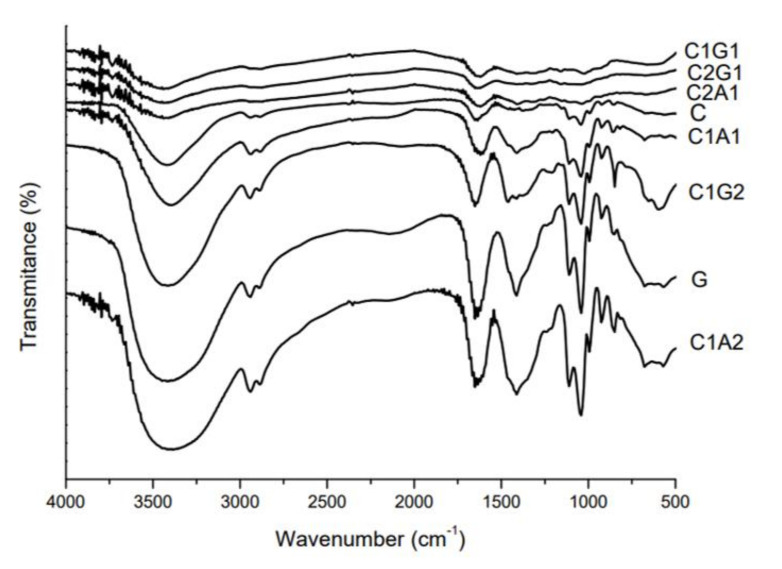
FTIR spectrum of SAH based on polymers and polyelectrolyte complexes. C—Chitosan; G—Xanthan gum; C1G1—Chitosan:Xanthan gum 1:1; C1G2—Chitosan:Xanthan gum 1:2; C2G1—Chitosan:Xanthan gum 2:1; C1A1—Chitosan:Alginate 1:1; C1A2—Chitosan:Alginate 1:2; C2A1—Chitosan:Alginate 2:1.

The interaction between biopolymers was also evaluated by FTIR. FTIR spectra for all formulations showed absorption peaks between 3700 and 2980 cm^−1^ (Figure 7), showing more intensity for G, C1G2, and C1A2. The 1650 cm^−1^ peak was present in C, G, C1G2, C1A1, and C1A2 samples. The decrease in intensity and hypsochromic shift of this peak to 1635 cm^−1^ in the FTIR spectra of C1G1, C2G1, and C2A1 were observed. For the samples containing higher amounts of chitosan (C2G1 and C2A1), as well as C1G1, the disappearance of peaks at 2945 and 2885 cm^−1^ and between 1413 and 1465 cm^−1^ was observed.

## 4. Discussion

The instability of the swollen structure of G and A after the “onion-like” process can be attributed to the electrostatic repulsion between polymer chains after the swelling process [16]. This swelling equilibrium is influenced by the presence of chitosan, which is in agreement with the results found by Costa-Júnior et al. [19]. They observed that the ability of hydrogel formation and the durability of its structural integrity were determined by the presence of chitosan in the formulations. The ability of chitosan to form hydrogels in aqueous solutions is a result of a nonpermanent network with physical interactions such as hydrogen or electrostatic bonds [20]. Costa-Júnior et al. [19] developed hydrogel based on chitosan and poly (vinyl alcohol covinyl acetate), chemically crosslinked by glutaraldehyde, showing a swelling degree of 150% to 700% in 24 h, in neutral solution. Our results demonstrate that when using natural polymers (chitosan and xanthan gum) and high pH to produce the complex, the swelling index of SAH was between 660% and 1000% at pH 7.0. Chitosan forms stable swollen hydrogels since its structure contains amino groups (cationic sites) that interact with the anionic group of other polyelectrolytes such as xanthan gum (glucuronic and pyruvic acid) and alginate [20].

When the SAH obtain process occurs at lower pH, SAH has decreased stability due to the formation of the interchain interactions preferentially. The material obtained in high pH favor interactions between the polymers. Therefore, the entanglements and secondary forces in this case can stabilize the hydrogel and hold water. These interactions are determined by the hydrophilic or hydrophobic nature of the polymeric chains and influence directly the behavior of the hydrogel swelling degree [19]. Xanthan gum presented the best chitosan interaction compared to alginate. The ionic complexation forms a strong intermolecular bonding between biopolymers that promotes the miscibility between the matrix components, ensures the decrease in solubility of the hydrogels, and consequently confers the potential storage of captured fluids.

MEV images showed that SAH with xanthan (C1G1 and C2G1) presented a more robust, thicker structure when compared to alginate samples (C1A1 and C2A1), and consequently with a better storage profile. Surface properties are important because they provide information regarding the wettability phenomenon in the material surface. SAHs were highly hydrophilic, since the water drop was instantaneously absorbed by the samples. The presence of a peak around 2θ = 20° in the X-ray diffraction results indicates the crystallinity of samples, determined mainly due to the presence of chitosan. These results agree with those reported by Qu et al. [20], who described that chitosan presents two crystalline peaks around 2θ = 10° and 20°. According to these authors, chitosan bonds with other compounds, such as lactic acid resulting in a new random rearrangement of polymeric chains in solution. This indicates that the original organized structure of chitosan is changed. As a consequence, this might result in the total amorphous conformation of chitosan linked to a new compound. The broader peak for a low proportion of chitosan samples (C1G2 and C1A2) was attributed to the absence of hydrogen bonding between amino groups and hydroxyl groups in the chitosan. We hypothesized that this interaction was broken by complexation process. Therefore, it may be associated with lower crystallinity of C1G2 and C1A2, due to the lower concentration of chitosan, and consequently, the lower solubility of the formulations.

The peaks around 3400 cm^−1^ at FTIR results are related to the stretching vibration of the –NH_2_ (chitosan samples) or –OH groups (alginate and xanthan samples) [21,22,23]. The high intensity of vibration in this region for G, C1G2, and C1A2 samples indicates that the hydrogen bonding is enhanced, since this peak is also related to the intramolecular hydrogen bonds present in the structure of all three polymers and polyelectrolytes. Changes in the intensity of C=O peak (around 1650 cm^−1^) are attributed to the overlapping of the peaks related to the amino groups of chitosan and the glucuronate groups of alginate and xanthan. The intensity of peaks is influenced by polymer interaction as well as the protonation state of polymers. At pH 7.0 (after the neutralization step of the “onion-like” process), the proportion of protonated and unprotonated amino groups from chitosan could have produced changes in the intensity of this peak. The hypsochromic shift of the C=O peak pointed to the interaction between NH_3_^+^ groups of chitosan and negative groups of xanthan or alginate [21]. In a similar study, Takara et al. showed that treatment of NaOH decreased the intensity of amino group peaks (around 1650 cm^−1^) in chitosan films due to deprotonation [24]. The disappearance of peaks related to C-H symmetric and asymmetric stretching (2945 and 2885 cm^−1^, respectively) and COO− (around 1400 cm^−1^) in C2G1, C2A1, and C1G1 samples corroborated the strongest interaction hypothesis in those polyelectrolytes [25].

## 5. Conclusions

The ionic polymeric interactions obtained at high pH following neutralization of the polyelectrolyte complex (“onion-like” methodology) were fundamental for the structural integrity of SAH after swelling, conferring better absorption potential and maintenance of structure. The NaOH treatment positively influenced the maintenance of the integrity of hydrogel after swelling for chitosan-based polyelectrolytes. We demonstrate that it is possible to obtain stable SAH after swelling from a polyelectrolyte complex based on biodegradable polymers. This material is suitable to be used in food and pharmaceutical industries—for example, as food packaging and disposable diapers.

## Figures and Tables

**Table 1:** Formulation of polyelectrolytes complexes.

Formulations	Polymers (w/v)		
	Chitosan (%)	Xanthan Gum (%)	Alginate (%)
C1G1	1.5	1.5	-
C1G2	1.0	2.0	-
C2G1	2.0	1.0	-
C1A1	1.5	-	1.5
C1A2	1.0	-	2.0
C2A1	2.0	-	1.0
C	3.0	-	-
G	-	3.0	-
A	-	-	3.0
